# Supplementary figures and images for: Reduced cutaneous CD200:CD200R1 signaling in psoriasis enhances neutrophil recruitment to skin
Source: Immun Inflamm Dis. 2022 Jun 6;10(7):e648. doi: 10.1002/iid3.648 (PMC9168552; doi:10.1002/iid3.648)

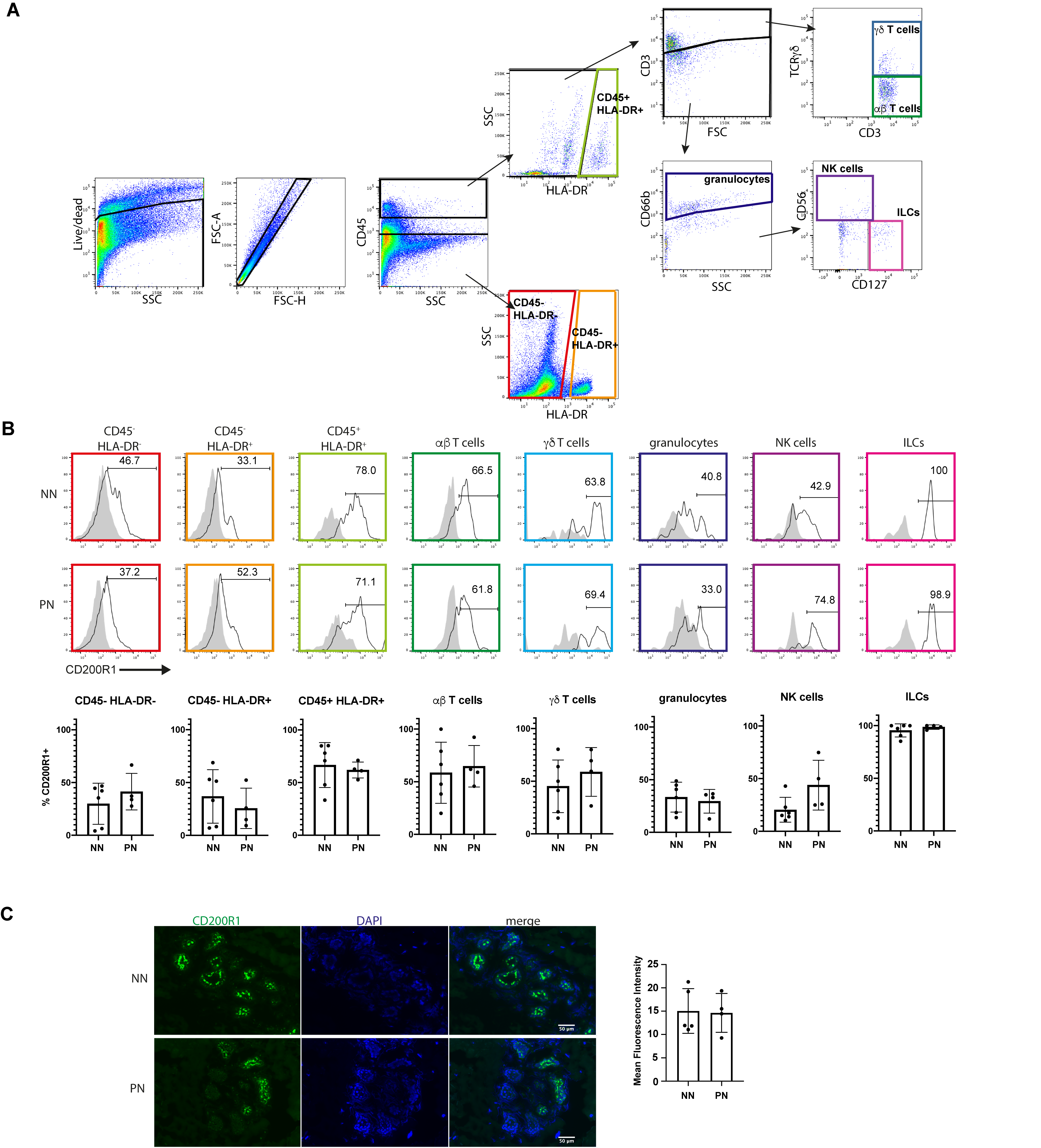

Supplement: Supplementary file 1 — Supporting information. [file IID3-10-e648-s001.tif]

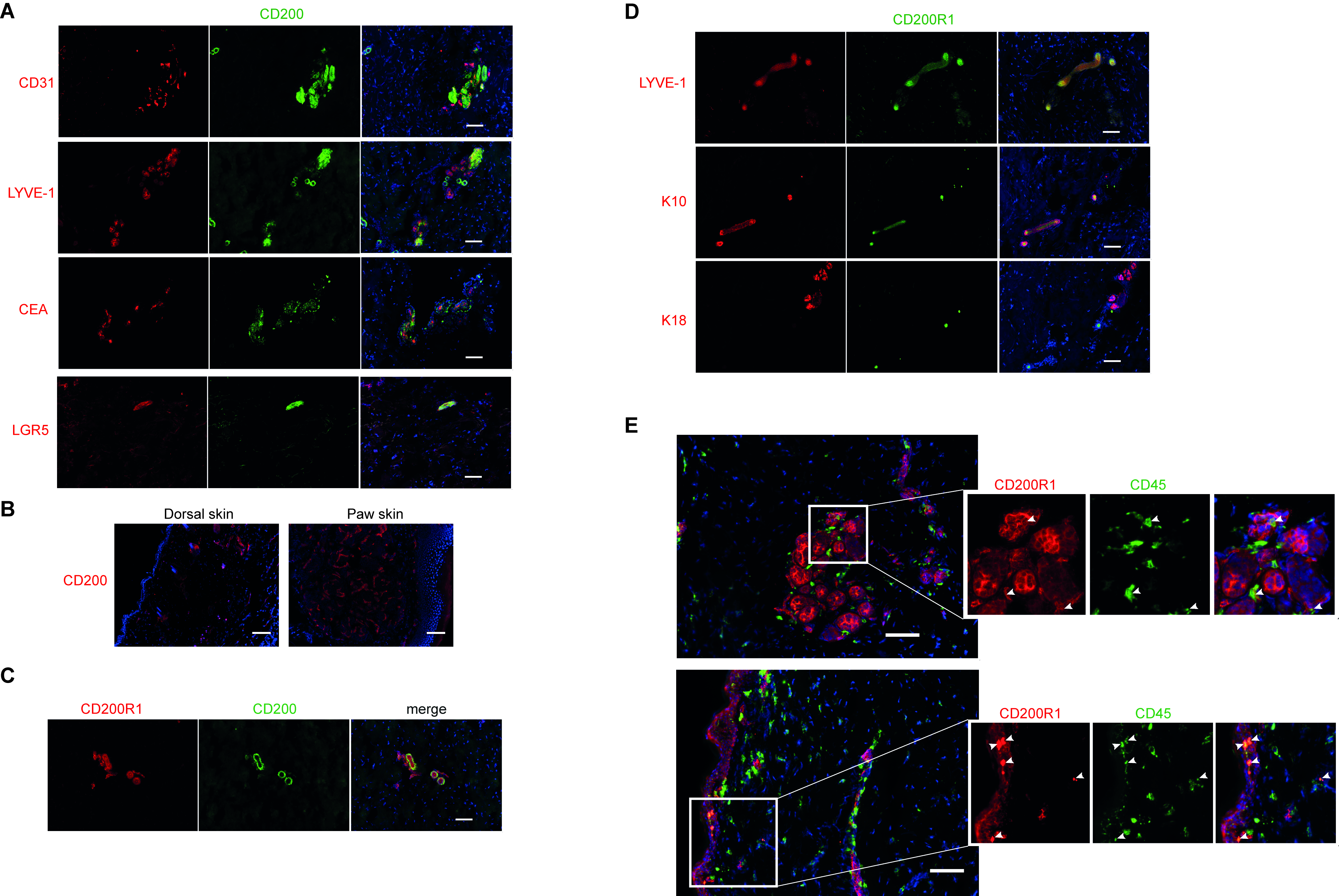

Supplement: Supplementary file 2 — Supporting information. [file IID3-10-e648-s002.tif]

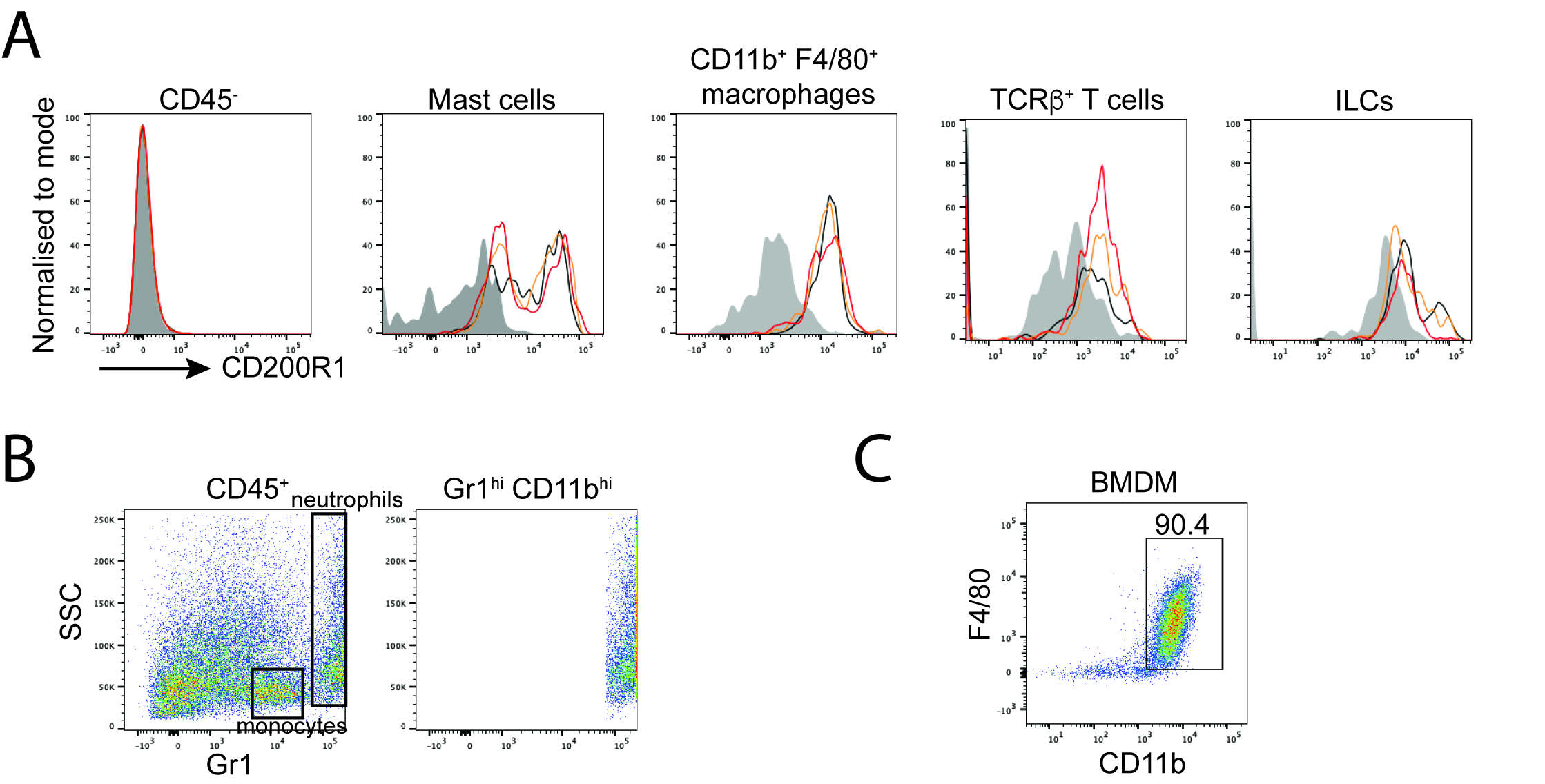

Supplement: Supplementary file 3 — Supporting information. [file IID3-10-e648-s004.tif]
